# Christ-Siemens-Touraine Syndrome with Self-mutilation Habit: An Unusual Presentation

**DOI:** 10.5005/jp-journals-10005-1044

**Published:** 2009-04-26

**Authors:** Gurusamy Kayalvizhi, R Neeraja

**Affiliations:** 1 Assistant Professor; 2 Assistant Professor, Department of Pedodontics and Preventive Dentistry, MR Ambedkar Dental College, Bangalore-560005 Karnataka, India

**Keywords:** Ectodermal dysplasia, Self- mutilation, Christ-Siemens-Touraine syndrome, Hypohidrotic, Hypodontia, Prosthetic treatment.

## Abstract

Ectodermal dysplasia exhibits a classic triad of hypohidrosis, hypotrichosis, and hypodontia. Self- mutilation could be due to organic or functional causes. The occurrence of selfmutilation with functional cause represents a diagnostic challenge to practitioners. In most of the instances dentists are the first to recognize patient with ectodermal dysplasia as they report primarily with a complaint of missing teeth. The most common type is hypohidrotic ectodermal dysplasia (Christ-Siemens-Touraine syndrome). A thorough knowledge of this disease with multidisciplinary approach aids in successful outcome of the treatment. This is an unusual case report of Christ-Siemens-Touraine syndrome with selfmutilation.

## INTRODUCTION

Ectodermal dysplasia (ED) is the congenital dysplasia of one or more ectodermal structures. Thurman published the first report of a patient with ED in 1848 but the term ectodermal dysplasia was later coined by Weech in 1929.ED’s are rare with their incidence estimated at 1 in 10,000 to 1 in 1, 00,000 births.^1^ The tissues primarily involved are the skin, hair, nails, eccrine glands and teeth. ED’s are congenital, diffuse, and nonprogressive. To date, more than 192 distinct disorders have been described. The most common is X-linked recessive Hypohidrotic Ectodermal Dysplasia (HED) also known as Christ-Siemens-Touraine syndrome.^2^ Self-mutilating behaviors are those in which the patient enjoys inflicting damage to himself. Prevalence of this condition in the general population is estimated to be around 750 in 1,00,000.^3^ Here we present a case of Christ- Siemens-Touraine syndrome with self- mutilation habit.

## CASE REPORT

**Fig. 1.**
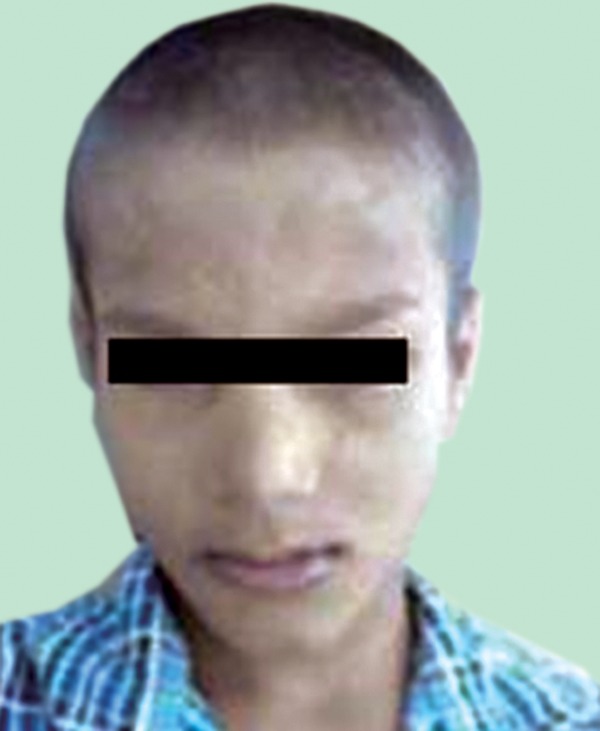
Frontal appearance of the patient

**Figs 2A to D:**
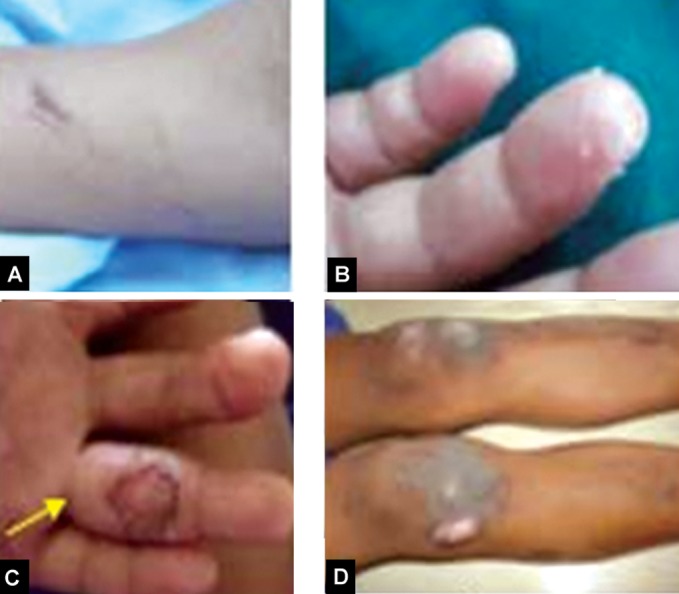
Self mutilation (A) Scratch marks over his hands, (B) Peeling of skin in fingers, (C and D) Burn marks on his palm and legs

**Fig. 3.**
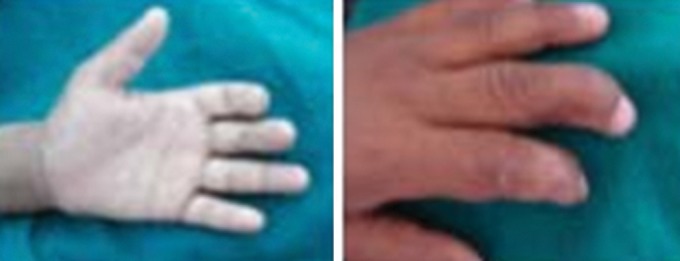
Hyperkeratosis of palm of hand with deformity of nails

A patient aged 7 years reported to our department with the chief complaint of missing teeth and difficulty in chewing food. Detailed history revealed that his parents had a consanguineous marriage, the patient was the second child in the family, and his birth was normal. Patient experienced high fever of unknown origin was intolerant to heat and did not sweat. Child was submissive, shy, and socially not interactive due to his appearance and loss of teeth. Medical history was not significant. Extraoral examinations revealed that his hair and eyebrows were sparse, his supraorbital ridges and chin were prominent, protuberant and everted lips with angular cheilitis and reduced lower facial height (Fig. 1) contributed to a senile facial expression. Skin was dry and rough. Scratch marks on his hands, peeling of skin in fingers, burn marks on palm of hand and legs, (Figs 2A to D) and multiple scars over his forehead were seen. Nails were deformed with hyperkeratosis of palms of his hands (Fig. 3).

**Figs 4A and B:**
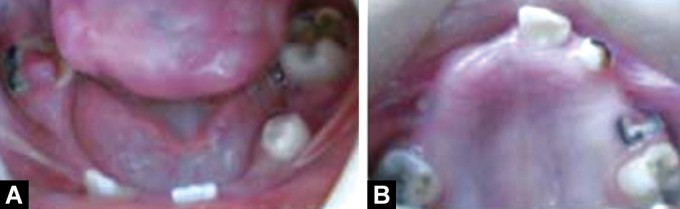
Intraoral view: (A) Lower arch, (B) Upper arch

**Fig. 5.**
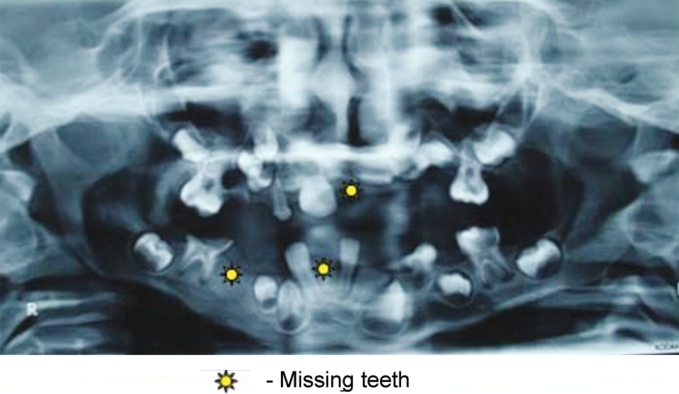
Orthopantomograph showing hypodontia

Intraoral examination revealed large tongue with depapillation in the anterior region giving it a "bald tongue" appearance. Thin alveolar crest was seen in the mandible whereas palate was normal. Following teeth were present (Figs 4A and B): 16, 55, 53, 11, 26, 31, 34, 36, 42 and 46. Parents were not aware of his missing primary teeth during primary dentition period. Radiographic evaluation by an orthopantomograph confirmed hypodontia with missing 21, 45, 41 and presence of tooth buds of remaining teeth with delayed root formation (Fig. 5).

Based on the history, pediatrician consultation, clinical examination, and radiographic findings it was diagnosed as Christ-Siemens-Touraine syndrome with self mutilation habit. Nervous tissue could have been affected in ectodermal dysplasia patients, but as nerve conduction test results in our patient were normal, it was diagnosed as self injurious habit. Patient was sent to a psychiatrist for psychological counseling.

**Fig. 6.**
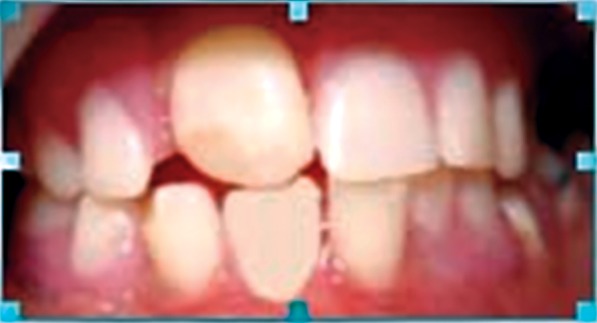
Postoperative view after removable partial denture insertion

**Figs 7A and B:**
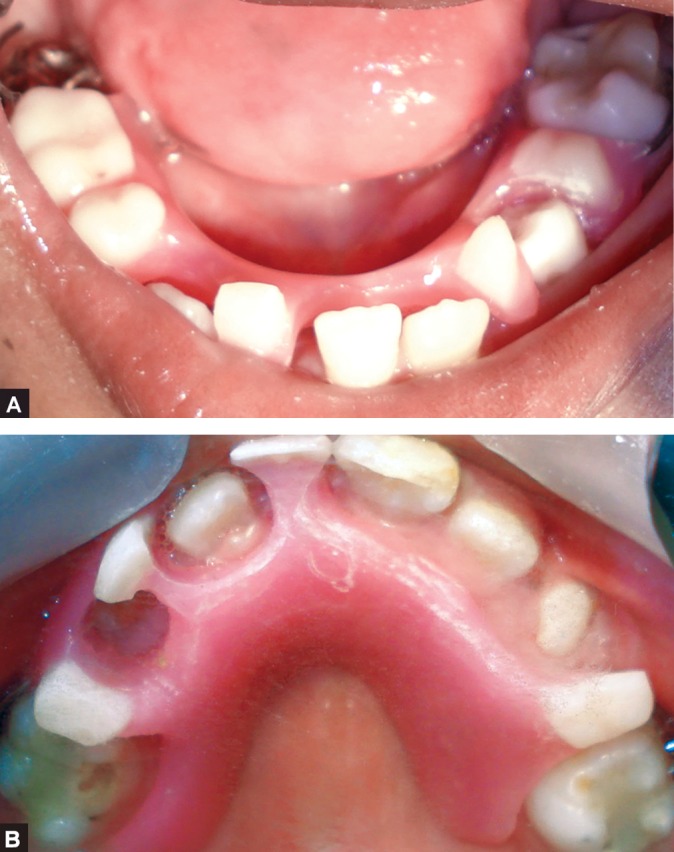
Follow-up after 1 year: (A) Lower arch, and (B) Upper arch

Treatment was planned following discussion with parent and patient. The dental treatment for ED patients is a challenge as treatment needs are life-long. Therefore, we planned preventive regimen by applying pit and fissure sealants for upper first permanent molars which had deep fissures and indirect pulp capping was done with respect to 36 and restored with composite resin. In pulpally involved 46, though it had an open apex root canal treatment was done and stainless steel crown was placed temporarily. Extraction of 53 and 55 were done. Enamel hypoplasia seen in 11 was treated by composite veneering. For prosthetic rehabilitation of the patient, diagnostic casts were made. To improve the child’s appearance, mastication, and speech, removable dentures were fabricated and inserted (Fig. 6). Occlusal interferences were eliminated. Post denture insertion instructions were given and patient was recalled after a week. On recall visit, it was observed that he had adapted well to the dentures and was happy about his appearance. Every three month recall visits were planned and one year follow up revealed the following erupted teeth- 12, 14, 22 and 32 (Figs 7A and B). Patient is still kept under observation.

## DISCUSSION

The term ED’s is a phenotypically heterogeneous group of disorders that affect tissues of ectodermal origin and occasionally those of non ectodermal origin. Many ED patients remain undiagnosed until infancy or childhood.^2^Our patient aged 7 years reported with the chief complaint of missing teeth, with thorough history, pediatric consultation, clinical and radiographic examination, it was diagnosed as Christ-Siemen-Touraine syndrome (HED -Hypohidrotic ectodermal dysplasia). The most characteristic findings of HED like hypotrichosis, hypodontia, hypohidrosis, dystrophic nails, reduced vertical dimension with angular cheilitis^4^ were noticed in our patient.

Differential diagnosis was performed to distinguish HED from sporadic oligodontia, radiotherapy in childhood, chondroectodermal dysplasia, cleidocranial dysplasia and other types of ED to confirm it as HED.^1^ It is usually an X linked recessive trait expressed only in men where women are carriers.^5^ Consanguinity increases the likelihood of expression of a trait or condition that is inherited in a recessive manner.^6^ Family history revealed consanguineous marriage of his parents. It can also occur in a family without any previous history of this syndrome. Though the gene for HED is identifiable by mutation analysis, it is done at very few laboratories at high cost, which was not affordable by our patient. Baldness of tongue could be due to nutritional deficiency, so he was advised to take nutritional supplements.

Nerve conduction test results in our patient were normal thus confirming self injurious habit due to psychological reasons. Self mutilation probably occurs more frequently than is realized because relatively few children will admit to the act unless they are observed practicing it. They may be incorrectly diagnosed.^6^ In our patient it was functional self mutilation due to emotional problem for which the patient was given psychological counseling.

Treatment of ED requires knowledge of growth and development, behavior management, fabrication of prosthesis, modification of existing teeth, motivation of patient and parent with long term follow-up.^7^ In our patient full mouth rehabilitation was done. In pulpally involved lower right molar root canal treatment was done and stainless steel crown placed. Vertical dimension of 1mm was increased when placing stainless steel crown, which was well tolerated by our patient. Enamel hypoplasia could have been due to ectodermal defect and nutritional deficiency.

Hypodontia is one of the major factors of ED and is almost always present.^1^ Our patient had 3 missing teeth with remaining permanent teeth being unerupted. Therefore, we planned prosthetic treatment using removable partial dentures, in order to improve patient appearance, mastication, speech and also to serve as a removable space maintainer. Prosthetic treatment was of great value to our patient from functional standpoint as well as for psychosocial reasons.^6^ After the prosthetic treatment and psychological counseling the patient became socially interactive and was happy with his appearance. Angular cheilitis seen due to reduced vertical dimension disappeared after prosthetic treatment. His nutritional status also improved.

Periodic recall check-up is an essential step in treating these patients.^1^ Replacement/modification of prosthesis is required due to continued growth and development of jaws and eruption of permanent teeth. Since our patient had few unerupted permanent teeth every 3 months recall visits were scheduled,^8^ so that dentures could be modified by drilling holes, thus making way for the erupting permanent teeth.^9^ In our case dentures were modified as and when the teeth erupted. Long-term follow-up is recommended in our patient till unerupted permanent teeth erupt and skeletal growth completes.

An early identification of ED is valuable as it increases the possibility to use growth adapted measures in the multidisciplinary treatment planning. Although, newer alternatives for rehabilitation like implants have been tried, placing them in children is of great concern.

## CONCLUSION

Ectodermal dysplasia has emotional consequence for affected individuals at early ages. Thus early clinical diagnosis and treatment planning of this disease is of great importance to restore mastication, speech, and esthetics. Self-mutilating habit due to psychological reasons might go unnoticed for this age group. So a proper history should be elicited. Periodic recall visits needs to be highlighted while planning treatment, as it presents a greater opportunity to modify prosthesis to accommodate ongoing growth and development of jaws.
